# Risk factors for recurrent venous thromboembolism in the European collaborative paediatric database on cerebral venous thrombosis: a multicentre cohort study

**DOI:** 10.1016/S1474-4422(07)70131-X

**Published:** 2007-07

**Authors:** Gili Kenet, Fenella Kirkham, Thomas Niederstadt, Achim Heinecke, Dawn Saunders, Monika Stoll, Benjamin Brenner, Christoph Bidlingmaier, Christine Heller, Ralf Knöfler, Rosemarie Schobess, Barbara Zieger, Guillaume Sébire, Ulrike Nowak-Göttl, the European Thromboses Study Group

**Affiliations:** a Israel National Haemophilia Centre, Sheba Medical Centre, Tel-Hashomer, Israel; b Neurosciences Unit, Institute of Child Health, University College London, London, UK; c Department of Child Health, Southampton General Hospital, Southampton, UK; d Institute of Clinical Radiology/Neuroradiology, University Hospital Münster, Germany; e Department of Medical Informatics and Biomathematics, University Hospital Münster, Münster, Germany; f Department of Paediatric Haematology/Oncology, University Hospital Münster, Münster, Germany; g Department of Radiology, Great Ormond Street Hospital, London, UK; h Leibniz-Institute for Arteriosclerosis Research, University of Münster, Münster, Germany; i Department of Haematology, Rambam Medical Centre, Israel; j Department of Paediatrics, University Hospital Munich, Germany; k Department of Paediatric Haematology/Oncology, University Hospital Frankfurt am Main, Germany; l Department of Paediatrics, University Hospital Dresden, Dresden, Germany; m Department of Paediatrics, University Hospital Halle, Halle, Germany; n Department of Paediatric Haematology/Oncology, University Hospital Freiburg, Freiburg, Germany; o Services de Neuropédiatrie, Université de Sherbrooke, Canada; p Université Catholique de Louvain, Louvain-La-Neuve Belgium

## Abstract

**Background:**

The relative importance of previous diagnosis and hereditary prothrombotic risk factors for cerebral venous thrombosis (CVT) in children in determining risk of a second cerebral or systemic venous thrombosis (VT), compared with other clinical, neuroimaging, and treatment variables, is unknown.

**Methods:**

We followed up the survivors of 396 consecutively enrolled patients with CVT, aged newborn to 18 years (median 5·2 years) for a median of 36 months (maximum 85 months). In accordance with international treatment guidelines, 250 children (65%) received acute anticoagulation with unfractionated heparin or low-molecular weight heparin, followed by secondary anticoagulation prophylaxis with low-molecular weight heparin or warfarin in 165 (43%).

**Results:**

Of 396 children enrolled, 12 died immediately and 22 (6%) had recurrent VT (13 cerebral; 3%) at a median of 6 months (range 0·1–85). Repeat venous imaging was available in 266 children. Recurrent VT only occurred in children whose first CVT was diagnosed after age 2 years; the underlying medical condition had no effect. In Cox regression analyses, non-administration of anticoagulant before relapse (hazard ratio [HR] 11·2 95% CI 3·4–37·0; p<0·0001), persistent occlusion on repeat venous imaging (4·1, 1·1–14·8; p=0·032), and heterozygosity for the G20210A mutation in factor II (4·3, 1·1–16·2; p=0·034) were independently associated with recurrent VT. Among patients who had recurrent VT, 70% (15) occurred within the 6 months after onset.

**Conclusion:**

Age at CVT onset, non-administration of anticoagulation, persistent venous occlusion, and presence of G20210A mutation in factor II predict recurrent VT in children. Secondary prophylactic anticoagulation should be given on a patient-to-patient basis in children with newly identified CVT and at high risk of recurrent VT. Factors that affect recanalisation need further research.

## Introduction

Although cerebral venous thrombosis (CVT) is associated with substantial mortality and morbidity,^1^, ^2^ its causes in children have not been intensively investigated, mainly because it occurs at a low frequency, estimated at 0·67 per 100 000 children.^1^ A wide range of underlying conditions^1^, ^2^, ^3^, ^4^, ^5^, ^6^, ^7^, ^8^, ^9^, ^10^, ^11^, ^12^ and symptoms at clinical presentation^1^, ^2^, ^3^ has been reported. Additionally, some subclinical factors, including prothrombotic disorders, are thought to contribute to presentation with the condition.^2^, ^7^, ^13^, ^14^, ^15^, ^16^, ^17^, ^18^, ^19^, ^20^, ^21^, ^22^, ^23^, ^24^, ^25^, ^26^, ^27^, ^28^

The long-term risk of a second cerebral or systemic venous thrombosis (VT) has received little attention, although overall proportions of such events were remarkably similar in two series with follow-up of 18 months and 12 months (13% and 12%).^1^, ^2^ The relevance of predisposing factors to the risk of recurrence, compared with other clinical and neuroimaging information, is not known. Partial or complete recanalisation has been documented in the majority of patients undergoing repeat venous imaging,^2^, ^26^ although 16% had persistent occlusion in both large series,^2^, ^26^ but the possibility that this problem is a risk factor for recurrent VT has not been assesed in children. Additionally, owing to the absence of appropriate randomised clinical treatment studies, guidelines for anticoagulation in children with VT are mainly adapted from adult protocols.^13^, ^29^, ^30^ We therefore investigated, in a European cohort study, the relevance of age at onset, underlying medical conditions, mode of administration of anticoagulation, neuroimaging data, and prothrombotic risk factors to a second VT in paediatric patients with a first CVT onset. The primary study objective was to calculate the symptomatic recurrence rate per 1000 person-years. Secondary aims were to calculate the time to recurrence, to examine the relevance of clinical, laboratory, and radiological risk factors to symptomatic recurrence, and to identify number needed to treat or to screen to prevent recurrence.

## Methods

### Study design and population

The multicentre follow-up study was done in accord with the ethics standards laid down in the updated version of the 1964 Declaration of Helsinki and was approved by the medical ethics committee of the University of Münster, Germany. Consecutively admitted term neonates and children with newly diagnosed CVT were enrolled in the German prospective cohort, with written parental consent. Appropriate research ethics approval was obtained from the institutional ethics review board at each site to include data of consecutively enrolled patients in England (anonymised clinical data from prospective stroke registries), Belgium, and Israel.

The present study was a multicentre cohort study to assess the rate of symptomatic VT recurrence per 1000 person-years after a first onset of CVT. The core protocol was developed by the German collaborative group and was adopted contemporaneously by centres in Belgium, England, and Israel; data were pooled across these sites to ascertain whether the results were generalisable, and to increase power for the secondary aims, which were to assess time to recurrence, predictors of recurrence, and number needed to screen for this relatively rare event. From July, 1996, to August, 2005, 396 patients from Belgium (n=5), Germany (n=299), Israel (n=53), and the UK (n=39) were analysed in the European paediatric database located in Münster, Germany. Consecutive patients with a first symptomatic CVT were recruited whether or not prothrombotic risk factors were present and recurrence was ascertained at follow-up in survivors. Preterm infants and patients older than 19 years at onset were excluded. First CVT was diagnosed locally by standard imaging methods—ie, Duplex sonography (neonates only), magnetic resonance imaging (MRI), and computed tomography (CT) or magnetic resonance venography and angiography.^1^, ^25^, ^26^, ^31^, ^32^

### Procedures

As previously described, bacterial or viral infections, head or vascular trauma, surgery, dehydration, immobilisation (bed rest >4 days) or obesity (body-mass index [BMI] >90th age-dependent percentiles^33^), jugular or subclavian central venous lines, solid tumours, leukaemia and lymphomas, anaemia, autoimmune diseases, renal diseases, metabolic disorders, birth asphyxia, and cardiac malformations were predefined as predisposing clinical conditions.^25^, ^26^, ^27^, ^28^, ^29^ Additionally, use of drugs such as steroids and *Escherichia coli* asparaginase, sympathomimetics, coagulation factor concentrates, or oral contraceptives, and nicotine abuse were classified as likely triggers. Patients who had CVT but did not show one of the criteria for a predisposing condition or trigger were classified as previously healthy.^25^

Patency of the cerebral veins in patients originally diagnosed with MRI and MR venography (MRV) was documented by use of MRI and MRV, 3–6 months after acute CVT onset, and was mainly classified centrally by one experienced paediatric neuroradiologist in Israel, Germany, or Belgium and the UK, on these radiological criteria:^1^, ^25^, ^26^, ^31^, ^32^ complete patency if no clot was present in the previously affected vessel; partial patency if the clot inside the lumen seemed to have decreased or shrunk; no change if the thrombus remained of similar size and extent in the same lumen; or extension if the clot extended to previously unaffected vessels. Recurrent CVT was diagnosed when MRI and MRV done in the acute phase of a new vascular accident showed fresh thrombotic material within a lumen of the vein—ie, a new intraluminal filling defect, compared with the results of previous tests. Incidental cases of apparent recurrence that were diagnosed without interim imaging while the patient was asymptomatic, and children in whom the first CVT was not confirmed by MRV, were excluded from analyses that assessed predictors of recurrent CVT.

VT in the deep veins of the leg and pulmonary embolism were diagnosed when standard imaging methods (ie, compression sonography, venography, CT, spiral CT, or MRI and perfusion lung scans for pulmonary embolism) in the acute phase of a new vascular accident showed fresh thrombotic material within a lumen of the vein.

According to published paediatric treatment guidelines for anticoagulation,^13^, ^29^, ^30^ patients were treated on an individual basis with low molecular weight heparin (LMWH), or unfractionated heparin followed by warfarin without knowledge of whether the patient was a carrier of prothrombotic risk factors or not. Long-term anticoagulation was defined as secondary prophylactic treatment with administration of anticoagulation for longer than 4 weeks.

Routine diagnostic tests done by the participating centres included face-to-face physical and neurological examination by an experienced paediatrician and assessment by standard imaging methods, at first thrombotic onset and at 8–12 weeks and 6 months later (before withdrawal of anticoagulation). Because of the limited availability and variability across participating centres, MRV was done to assess follow-up patency rates within 3–6 months. In children with clinically suspected CVT recurrence, MRI and MRV were done within a 48-h window (allowing for unavailability at weekends) after emergency CT, immediately after hospital admission. Local reports were accepted for emergency imaging at initial presentation, but for the majority of follow-up MRI and MRV procedures, independent reading was available from the study centres (London, Tel Hashomer, and Münster). Since very few cases occurred in Belgium, data for this country were analysed together with those from the UK.^2^ After discontinuation of anticoagulation, asymptomatic patients were followed-up every few months for the first year and at longer intervals thereafter (at least yearly). All patients were seen at least once for a follow-up with a paediatric neurologist.

Laboratory analyses for thrombophilic factors associated with risk of VT in children, including those for the presence of the G1691A mutation in factor V, the G20210A mutation in factor II, antithrombin, protein C, protein S, and antiphospholipid antibodies or lupus anticoagulants were done in the participating study centres for 367 patients, as previously described.^2^, ^22^, ^23^, ^26^, ^27^ Analysis of lipoprotein (a), reported to be a risk factor for arterial stroke in children in 1997,^14^, ^23^ was undertaken in 200 patients.

### Statistical analyses

For the primary study objective, to calculate the symptomatic recurrence rate per 1000 person-years, based on the recruitment period of 9 years and a median follow-up period of 36 months, the sample size was n=384 (survivors) with 22 symptomatic recurrences diagnosed. Predictors that could possibly affect the study outcomes, defined a priori on the basis of published data, were: age at onset, administration of anticoagulants before relapse (relapse on anticoagulation treatment), patency rates, and prothrombotic risk factors significantly associated with a first VT onset.^1^, ^13^, ^18^, ^21^, ^22^, ^23^, ^24^, ^25^, ^26^, ^27^, ^28^, ^29^, ^30^, ^31^, ^32^, ^33^, ^34^, ^35^ Using a rule of thumb for proportional hazards analysis including about ten outcomes for each independent predictor,^36^ the final statistical model included three predictors, which were obtained from results of univariate survival analysis (first step). A second step calculated the possible effect of three prothrombotic risk factors—factor V G1691A mutation, factor II G20210A mutation, and raised lipoprotein (a)—on recurrent VT. Significant predictors from both steps were included in the final multivariate analysis. Model assumptions and goodness-of-fit were assessed using the Hosmer and Lemeshow goodness-of-fit test (SAS version 8.3).^37^ As binary outcomes of interest, anticoagulation treatment before relapse (yes, no), persistent venous occlusion (yes, no), and presence of factor II G20210A mutation (thrombophilic gene mutation known to increase risk of VT in children;^22^ yes, no) were incorporated in the model. Further statistical analyses were done with Stata (version 8.0) and StatView 5 software (SAS Institute). The recurrence rates were calculated as the number of recurrent VTs per 1000 person-years. The secondary study objective, time to recurrence, calculated as the probability of thrombosis-free survival as a function of time, was assessed by Kaplan-Meier univariate analysis. The log-rank test was used to test for differences in thrombosis-free survival between groups. Patients were censored from the survival analysis at either death unrelated to VT recurrence or loss to follow-up, using data from the last clinical follow-up visit. To evaluate an independent contribution to the risk of recurrent VT and to adjust for potential confounders, the hazard ratio (HR) and 95% CI were estimated from Cox's proportional hazards model. Possible interaction effects between predictors were assessed with the Wald test (SAS version 8.3). To further test the overall hypothesis of no effect of all predictors the likelihood ratio test was done. Continuous data are presented as median (range) and were assessed by non-parametric statistics with the Wilcoxon-Mann-Whitney U test or Kruskal-Wallis test. To compare frequency distributions of fatal outcome, the χ^2^ test or Fisher's exact test was used. In a subset of German patients the degree of agreement beyond chance between local and central readers were measured with the κ statistics (Stata 8.0). The criterion for statistical significance was set at α=0·05. p values were based on two-sided tests. Numbers needed to treat and numbers needed to screen were calculated as previously described.^38^

### Role of the funding source

The funding sources of the study had no role in study design, data collection, data analysis, data interpretation, or in writing of the report. The corresponding author had access to all data and had final responsibility for submitting the report for publication.

## Results

Table 1 summarises patients' characteristics at first thrombotic onset. MRI was undertaken at initial presentation in 379 (95%) cases. 12 children died within 2 weeks of presentation with a first episode of CVT, one of whom had received anticoagulation. Causes of deaths were severe infection in three, intracranial bleeding in two, and brain herniation after severe brain oedema in seven cases. The risk of a second VT was assessed in 384 consecutively recruited children aged neonatal to 18 years (median 5·2 years; 236 boys [60%]) who survived a first episode of CVT, and who were followed up for a median time of 36 (range 0·1–85) months. Detailed data of the follow-up group are summarised in table 2. When comparing children aged 2 years or younger with those older than 2 years, we noted no significant differences with respect to sinuses involved at first thrombotic onset (p=0·164) and methods of treatment used (p=0·657). Additionally, we found no significant difference between the groups with respect to imaging methods used (p=0·511).

**Table 1:** Clinical characteristics of CVT study population at thrombotic onset

		**Israel (n=53)**	**Germany (n=299)**	**UK/Belgium (n=39/5)**	**Total (n=396)**
**Population at first CVT onset**					
White		46 (87%)	299 (100%)	37/5 (95%/100%)	387 (98%)
Arab		7 (13%)	0	0	7 (2%)
Afro-Caribbean		0	0	2/0 (5%/0%)	2 (1%)
Male		28 (56)	180 (60)	28 (64%)	236 (60%)
Median age, years (range)		3 (0·01–17·7)	6 (0·01–17·9)	6 (0·1–14)	5 (0·01–17·9)
Number of neonates		9 (17%)	66 (22%)	0	75 (19%)
**Venous sinuses involved at onset, infarction, haemorrhage**					
Superficial					
	Sagittal	25 (47%)	153 (51%)	10 (23%)	188 (47.5%)
	Transverse	10 (19%)	34 (11%)	2 (4.5%)	46 (12%)
	Sigmoid	5 (10%)	21 (7%)	1 (2.3%)	27 (7%)
Deep venous system					
	Deep veins	1 (2%)	3 (1%)	0	4 (1%)
	Straight sinus	1 (2%)	2 (0.7%)	1 (2.3%)	4 (1%)
	Cavernous	0	2 (0.7%)	5 (11%)	7 (2%)
Other locations					
	Cortical veins	0	1 (0.3%)	2 (4.5%)	3 (1%)
	Multiple (>2 sinuses involved)	11 (22%)	83 (28%)	23 (52%)	117 (30%)
Infarction on initial imaging		8 (15%)	25 (8%)	12 (27%)	37 (9%)
Haemorrhage on initial imaging		3 (6%)	18 (6%)	12 (27%)	33 (8%)
**Anticoagulation, underlying conditions, acute deaths**					
Anticoagulation withheld because of haemorrhage on initial imaging		2 (4%)	15 (5%)	6 (13%)	23 (6%)
Underlying medical conditions present		34 (68%)	183 (61%)	23 (53%)	240 (61%)
Deaths after CVT onset		3 (6%)	5 (2%)	4 (9%)	12 (3%)
Acute anticoagulation given		48 of 53 (91%)	185 of 299 (62%)	17 of 44 (39%)	250 of 396 (63%)
	LMWH	32 (68%)	92 (50%)	3 (18%)	128 (51%)
	Unfractionated heparin	15 (32%)	93 (50%)	14 (82%)	122 (49%)
	In patients who died immediately	1	0	0	1 of 12 (8%)

Data are number (%) unless otherwise stated. Cohort data previously published in part.^2^, ^26^, ^27^

**Table 2:** Clinical characteristics of surviving population followed up for recurrence

			**Israel**	**Germany**	**UK/Belgium**	**Total**
Surviving CVT population			50 (94%)	294 (98%)	40 (91%)	384 (98%)
Recurrences			2 of 50 (4%)	15 of 294 (5%)	5 of 40 (13%)	22 of 384 (6%)
	In superficial sinuses					
		Sagittal*	2 (100%)	2 (13%)	1 (20%)	5 (23%)
		Transverse*	0	3 (20%)	0	3 (14%)
	In deep venous system					
		Deep veins	0	1 (7%)	0	1 (4.5%)
		Straight sinus	0	2 (13%)	0	2 (9%)
	At other locations					
		Cortical veins	0	2 (13%)	0	2 (9%)
		VT/pelvic	0	2 (13%)	4 (80%)	6 (27%)
		Pulmonary embolism	0	2 (13%)	0	2 (9%)
		Intracardiac	0	1 (7%)	0	1 (4.5%)
		Multiple thromboses: VT and sinuses*	0	2 (13%)	0	2 (9%)
Median follow-up, months (range)			45 (6–85)	36 (6–72)	13 (0·1–48)	36 (0·1–85)
Deaths after CVT recurrence			1 (50%)	0	1 (20%)	2 (9%)
Prophylactic anticoagulation >6 months			39 of 50 (78%)	125 of 294 (43%)	1 of 40 (3%)	165 of 383 (43%)‡
	LMWH		25 (64%)	101 (81%)	0	126 (76%)
	Vitamin K-antagonists		14 (36%)	25 (20%)	1 (3%)	40 (24%)
Median (range) anticoagulation duration, months			6 (0–84)	3 (0–48)	24	6 (0–84)
3–6 months patency†/repeat MRV done			45 of 50 (90%)	200 of 294 (68%)	21 of 40 (53%)	266 of 383 (69%)‡
	Complete		26 (58%)	89 (45%)	8 (38%)	123 (46%)
	Persistent occlusion		2 (5%)	24 (12%)	4 (19%)	30 (11%)
	Partial		17 (38%)	87 (44%)	9 (43%)	113 (42%)

Data are number (%) unless otherwise stated.

* Combination of sagittal, superior and transverse sinus.

† Confirmed by MRI/MRV in patients who had at least one MRV before recurrence.

‡ n=383 (not 384) because of one early death after first CVT onset.

**Figure 1:**
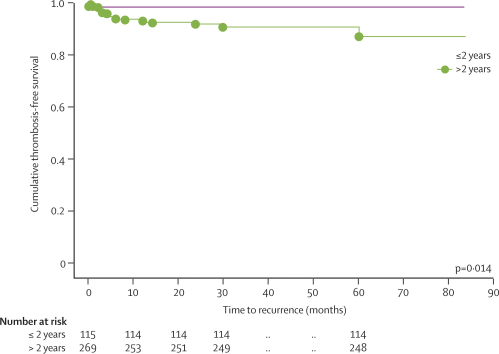
Cumulative thrombosis-free survival with respect to age at first thrombosis onset

A second VT event occurred in 22 of 384 surviving children at a median of 6 months (range 0·1–85) after the first event, and at a median age of 13 years (range 2·5–16·2), with no significant difference between countries in the proportion with a second event (table 2; p=0·309). The recurrence rate per 1000 person-years was 21·2 (95% CI 13·9–32·1) for the entire cohort and 29·1 (18·9–44·7) for children older than 2 years. At recurrence, immediate death occurred in two of 22 cases due to haemorrhage with severe brain oedema and herniation. The distribution of recurrent VT and the VT-related death rates recorded are shown in table 3. Five children died due to their basic disease after the end of the median follow-up period (36, 38, 47, 48, and 50 months after first CVT onset). About 70% of recurrent VT events occurred within the first 6 months after the initial CVT. Recurrent VT occurred within the veins of the brain in 13 of 22 cases (59%); deep venous thrombosis of the legs and pelvis was diagnosed in eight cases (in two cases associated with CVT recurrence, table 2), pulmonary embolism in two, and intracardiac thrombosis in one. A bivariate analysis showed no significant difference between recurrence of CVT and VT in other locations with respect to the predictor variables chosen (persistent venous occlusion [CVT] p=0·966; anticoagulation before relapse p=0·146; factor II G20210A mutation p=0·999). Brain lesions at second VT were documented as new thrombus formation in previously unaffected segments, a new vascular filling defect of the internal cerebral veins, cortical veins, or within the contralateral transverse sinus. No child aged 2 years or younger with initial CVT showed recurrent VT during the follow-up period. The youngest child with recurrent VT had the first CVT event aged 25 months. Compared with the average age of the entire study cohort (5·2 years) the median age of patients who had a recurrence was 13 years. Cumulative thrombosis-free survival with respect to age at first thrombosis onset is presented in figure 1; the log-rank test showed a significant difference between the two curves.

**Table 3:** Timing (months after first CVT onset) of VT recurrences in 384 surviving patients with CVT

	**Number of patients with recurrences (%)**	**Cumulative number of patients with recurrences (%)**	**Cumulative number of patients without recurrent VT or death (%)**
<1	4 (1·0%)	4 (18%)	380 (99%)
1 to <3	6 (1·5%)	10 (45%)	374 (97%)
3 to <6	5 (1·3%)	15 (68%)	369 (96%)
6 to <12	2 (0·5%)	17 (77%)	367 (96%)
12 to <24	3 (0·8%)	20 (91%)	364 (95%)
24 to <36	1 (0·3%)	21 (95%)	363 (95%)
>36	1 (0·3%)	22 (100%)	362 (94%)

Before recurrent VT, 17 of 22 children affected (77·3%) were suffering from chronic, relapsing, or recurrent medical conditions: acute lymphoblastic leukaemia relapse (five), lymphoma (two), brain tumour (one), head trauma (one), vascular malformation (one), type 1 diabetes (one), relapse of nephrotic syndrome (two), infectious diseases (three), and heparin-induced thrombocytopenia type 2 (one); additionally, two adolescent girls were taking oral contraceptives. Rethrombosis occurred in three previously healthy individuals on no medication.

Acute anticoagulation was undertaken with LMWH or unfractionated heparin in 250 children (65%), and 165 patients of the entire cohort (43%) received continuous secondary anticoagulant prophylaxis: LMWH was given in 80% (126) of cases over a median period of 6 months (range 3–12), and 20% received warfarin over a period of 12 months (6–84). Although all the other predictors (table 1) and rate of VT recurrence were similar in the participating countries (table 2), patients enrolled from the UK had a significantly lower rate of anticoagulant administration compared with children recruited from Israel and Germany (p=0·002). The distribution of infarction and haemorrhage on initial imaging, and the proportion of patients in whom anticoagulation was withheld due to bleeding symptoms at onset of CVT are shown in table 1. On unfractionated heparin, one adolescent girl developed heparin-induced thrombocytopenia type 2. One neonate on LMWH died with intracranial haemorrhage; one fatal intracerebral haemorrhage occurred in a child with intractable nephrotic syndrome anticoagulated with unfractionated heparin for recurrent CVT, and minor bleedings from puncture sites were documented in two further children treated with LMWH during the first CVT onset. No other child with antithrombotic therapy had a major haemorrhage subsequently.

Only six of 22 patients (27%) were on secondary anticoagulation immediately before recurrent VT. Besides the adolescent girl who had heparin-induced thrombocytopenia type 2 on unfractionated heparin, three of the five children on prophylactic LMWH therapy showed reduced 4-h anti-factor Xa activity (0·1, 0·25, and 0·34 IU/mL) at the time of recurrence. The other two children who relapsed on LMWH had adequate anti factor Xa activity.

When comparing the treatment with the non-treatment group by univariate analysis, recurrence was commoner in children with underlying medical conditions than in those with idiopathic VT, but the difference was not significant (p=0·124). In addition, when comparing the two treatment groups we noted no increase in the risk of recurrence for children in whom thrombophilia was diagnosed (p=0·852), or in children with the combination of a medical condition and prothrombotic disorder (p=0·734).

**Figure 2:**
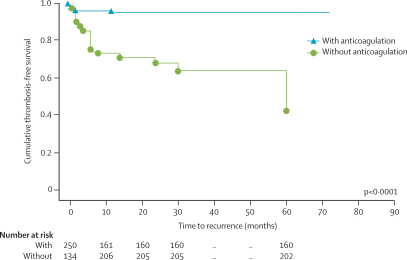
Cumulative thrombosis-free survival in children with and without anticoagulation before recurrent VT

In figure 2, the survival curves in children on anticoagulant administration compared with patients without anticoagulant before relapse are shown. Non-administration of anticoagulant in clinical situations that increase the risk of venous thrombosis—such as infections, dehydration, malignant disease, immobilisation, relapse of acute lymphoblastic leukaemia or nephrotic syndrome, and in children with idiopathic CVT (n=3) was significantly associated with an increased risk of recurrence (log-rank test p<0·0001, figure 2).

In addition to the 22 children with symptomatic recurrences, follow-up venography was undertaken in 244 surviving children who remained free of clinical recurrence. Thus, 69% of cases had follow-up imaging, with 250 of 266 (94%) confirmed centrally by experienced neuroradiologists in the three study centres (Israel n=30; Germany n=203; Belgium and UK n=17). Patency was judged in children who had undergone MRV at CVT onset only. Our calculations showed substantial agreement (89·1%), beyond that expected by chance alone (41·1%), between local and central readers in the subset of 84 German patients tested (κ=0·82; 95% CI 0·70–0·93; p<0·0001).

**Figure 3:**
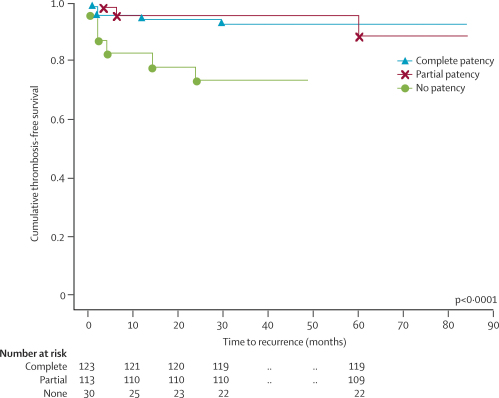
Cumulative thrombosis-free survival in children with complete, partial, and no patency at 3–6 months Analysis included only patients for whom full patency data were available (n=266); in the other patients, early second events occurred before completion of patency follow-up. Recurrences in children with full data: four complete, three partial, eight none.

Compared with those with complete or partial patency of the initial sinuses involved, children with no patency at follow-up venous imaging had a shorter thrombosis-free survival for CVT and non-CVT recurrence (table 4). The combined data for CVT and VT recurrences are shown in figure 3.

**Table 4:** Three-step Cox proportional hazards model

		**HR (95% CI)**	**p**
**First step (ref: anticoagualation before relapse, any recanalisation)**			
Age at onset (months)		1·0 (0·99–1·01)	0·090
No anticoagulation before relapse		16·1 (4·3–60·9)	<0·0001
Persistent CVT occlusion			
	CVT and VT recurrence	5·1 (1·6–16·1)	0·005
	CVT recurrence alone	5·4 (1·3–22·8)	0·019
	VT recurrence alone	9·1 (1·7–49·5)	0·010
**Second step (ref: factor II wild type, FV wild type, lipoprotein (a) <300 mg/L**			
Factor II G20210A		5·5 (1·5–20·8)	0·011
Factor V G1691A		1·9 (0·2–14·9)	0·530
Lipoprotein (a) >300 mg/L		0·9 (0·3–3·0)	0·868
**Third step (ref: anticoagulation before relapse, any recanalisation, factor II wild type)**			
No anticoagulation before relapse		11·2 (3·4–37·0)	<0·0007
Persistent CVT occlusion		4·1 (1·1–14·8)	0·032
Factor II G20210A		4·3 (1·1–16·2)	0·034

Multivariate analysis was done in three steps. The first included the significant predictors derived from univariate Kaplan-Meier analysis—ie, age at first CVT onset, non-administration of anticoagulant before relapse, and persistent venous occlusion (table 4). The second analysis included three potential prothrombotic risk factors. Cox regression analysis showed that the heterozygous factor II G20210A mutation was a significant risk factor for recurrent VT in the present cohort (table 4). The heterozygous factor V G1691A mutation and raised lipoprotein (a) were not associated with an increased risk of recurrent VT. Additionally, no recurrent VT was diagnosed in two children with antithrombin deficiency, in two with protein C deficiency, or in five in whom persistent anticardiolipin antibodies or lupus anticoagulants were reported. According to the current standard of care, children with persistent anticardiolipin antibodies or lupus anticoagulants were on long-term anticoagulation with warfarin.^30^ The third proportional hazards analysis, which included significant predictors from the first two steps, showed that non-administration of anticoagulation before relapse, persistent venous occlusion, and being heterozygous for G20210A mutation in factor II were independent risk factors for recurrent VT in the cohort (table 4). Possible interaction effects between predictors were assessed with the Wald test: p values were 0·636 for persistent venous occlusion and factor II G20210A mutation, 0·707 for previous anticoagulation and factor II G20210A mutation, and 0·982 for persistent venous occlusion and previous anticoagulation. The likelihood ratio test, used to further test the overall hypothesis of no effect of all predictors in the final model, gave a p value of <0·0001. Since no comparable paediatric CVT cohort was currently available to validate the hypothesis derived from the final statistical model, we did an internal validation. Results of the Hosmer-Lemeshow goodness-of-fit test (p=0·586) gave evidence that the final model predicts outcome in the cohort investigated.

The number needed to screen to detect at least one factor II G20210A carrier was 16, the overall number needed to treat to prevent one recurrent VT was 32 for the whole group and three in carriers of the factor II mutation who were older than 2 years at first thrombotic onset.

## Discussion

In this group of paediatric CVT patients from three European registries, the overall yearly rate of a second VT event was 22·2 per 1000 person-years within a median period of 6 months after first VT onset. Our data are in accord with findings on cerebral vein and dural sinus thrombosis in adult patients;^39^, ^40^, ^41^, ^42^, ^43^ the cumulative recurrence rate of either CVT or any other thrombotic event in these studies ranged from 0% to 26%. Ferro and colleagues reported in their series of 624 adults that 58·8% of patients were not under any anticoagulant treatment at time of recurrence.^41^ In these studies, recurrent events occurred over a mean follow-up period of 18–77 months in patients without anticoagulant at the time of recurrence who had previously completed a standard 3–6 months course of anticoagulant treatment,^39^, ^40^, ^41^, ^42^, ^43^ but the effect of patency rates on recurrence was not addressed.

Our survival analysis suggests that in children older than 2 years, failure to recanalise and the presence of the G20210A mutation in factor II are risk factors for recurrent VT. Interestingly, failure to recanalise predicted CVT and non-CVT recurrence when both were assessed independently. A possible mechanism affecting thrombus formation and clot patency rates is the stability of the clot itself, which might depend not on the anatomical venous location but on other factors, including genetic polymorphisms, for example in the fibrinogen or the factor XIII gene. Iron deficiency might also play a part in failure to recanalise,^2^ which might be a reason why this factor affects risk of recurrence independently of anticoagulant treatment; we could not confirm such an association, because we did not have these data for most patients. Our observations about common thrombophilias were in line with those of a meta-analysis in adults, which showed an estimated population-attributable risk for recurrence of 6·7 (95% CI 3·4–9·9) for the factor II G20210A variant, but could not confirm this risk for the heterozygous factor V G1691A mutation, with an estimated population attributable recurrence risk of 9·0 (95% CI 4·5–13·2) in adult patients with VT.^44^

Our data also show an increased risk for recurrent VT in children with a first CVT onset after age 2 years, with a recurrence rate per 1000 person-years in accord with data reported in adult European patients with CVT.^39^, ^40^, ^41^, ^42^ Additionally, our results provide evidence that the use of anticoagulants should be considered on an individual patient basis in clinical situations where the risk of venous thrombosis is increased. The prolonged use of such drugs in a physically active age group must be weighed against the risk of haemorrhage. Since the number needed to treat to prevent one recurrent VT in this cohort was 32, prolonged anticoagulation might only be justified in children older than 2 years who also carry the factor II mutation (number needed to treat=three), in those with idiopathic CVT—ie, with no pre-existing illness or triggers, or in children in whom recurrent VT has already occurred. Practically, rather than receiving indefinite anticoagulation, children with chronic conditions associated with VT might require such treatment only in high-risk situations—eg, postoperatively, if prolonged immobilisation is needed, or if the patient becomes dehydrated.

The rate of recurrent thrombosis over a median follow-up period of 36 months reported here is lower than that previously reported in children,^1^, ^2^ probably because prolonged anticoagulation was undertaken in 65% of the German and Israeli patients over a period of at least 6 months, whereas in the UK-Belgian and Canadian studies only 40% and 53% of patients, respectively, received antithrombotic therapy over the first 3 months.^1^, ^2^

We report a low rate of recurrence in children aged 2 years and younger, and a median age of 13 years at second VT in children older than 2 years at onset. Since the younger children all passed the first risk period of childhood thrombosis—ie, the first year of life—we propose that peculiarities of developmental haemostasis associated with the well-known second peak of VT risk during puberty and adolescence, via hormonal changes and a downregulation of the fibrinolytic system, contribute to the increasing recurrence rate reported here.^45^ This proposal is supported by Goldenberg and colleagues'^35^ finding that children with deep venous thrombosis are more likely to have a recurrence during puberty.

The potential biases due to including only data from centres with an interest in paediatric CVT, non-randomisation of treatment methods, and use of different imaging methods to diagnose CVT at first onset, are limitations of our follow-up study. A larger international treatment study is needed to answer the open questions. The mode of anticoagulation which in accord with published paediatric guidelines was recommended by the treating physicians, was the major source of heterogeneity of the pooled patient cohorts. Anticoagulation regimens recommended for children and known to act on an equivalent basis in adult patients with VT were used.^29^, ^30^ Patients recruited from the UK had a significantly lower rate of anticoagulant administration than children recruited from Israel and Germany, but the prevalences of the other factors used to predict recurrence were similar. Based on the work of Revel-Vilk and coworkers,^34^ who showed in an independent cohort that VT patency at follow-up in children with venous thromboses was not related to anticoagulation therapy, and supported by our finding of no significant differences between the recurrence rates in the three study countries, we believe that pooling of the three registries was a valid way to address the important question of recurrence risk in relation to anticoagulation. However, further work is needed. Central reading of acute and follow-up imaging for all patients with assessment of interobserver variability would be an important component of a prospective study, which would ideally be population-based. The data obtained from our final model should also be validated in a different cohort of patients with a first onset of CVT; the opportunity to undertake such a study in partnership with the International Paediatric Stroke Study collaboration is currently under investigation. The strength of this study is that in the large European cohort, the rate of recurrent VT after CVT in children was comparable between different countries, which allows us to better calculate the number of patients that need to be enrolled to sufficiently power a randomised treatment study. Until data from such studies are available, the message of this follow-up study is clearly that administration of secondary anticoagulation prophylaxis should be considered on an individual patient basis in children with newly identified CVT in situations where the risk of VT is high.
